# Electroencephalography (EEG) and the Quest for an Inclusive and Global Neuroscience

**DOI:** 10.1111/ejn.70078

**Published:** 2025-03-18

**Authors:** Faisal Mushtaq, Agustín Ibáñez

**Affiliations:** ^1^ School of Psychology University of Leeds Leeds UK; ^2^ Leeds Institute for Data Analytics University of Leeds Leeds UK; ^3^ NIHR Leeds Biomedical Research Centre Leeds UK; ^4^ Latin American Brain Health Institute Universidad Adolfo Ibañez Santiago Chile; ^5^ Global Brain Health Institute (GBHI), University of California, San Francisco US and Trinity College Dublin Dublin Ireland; ^6^ Cognitive Neuroscience Center Universidad de San Andrés Buenos Aires Argentina; ^7^ Trinity College Dublin University of Dublin Dublin Ireland

**Keywords:** community engagement, data harmonization, electroencephalography (EEG), global neuroimaging, inclusivity

## Abstract

The current lack of diversity in neuroimaging datasets limits the potential generalisability of research findings. This situation is also likely to have a downstream impact on our ability to translate fundamental research into effective interventions and treatments for the global population. We propose that electroencephalography (EEG) is viable for delivering truly inclusive and global neuroscience. Over the past two decades, advances in portability, affordability, and computational sophistication have created a tool that can readily reach underrepresented communities and scale across low‐resource contexts—advantages that surpass those of other neuroimaging modalities. However, skepticism persists within the neuroscience community regarding the feasibility of realizing EEG's full potential for studying the brain on a global scale shortly. We highlight several challenges impeding progress, including the need to amalgamate large‐scale, harmonized datasets to provide the statistical power and robust computational frameworks necessary for examining subtle differences between populations; the advancement of EEG technology to ensure high‐quality data acquisition from all individuals—irrespective of hair type—and operable by nonspecialists; and the importance of engaging directly with communities to cocreate culturally sensitive and ethically appropriate research methodologies. By tackling these technical and social challenges and building on initiatives dedicated to inclusivity and collaboration, we can harness EEG's potential to deliver neuroscience genuinely representative of the global population.

## Graphical abstract

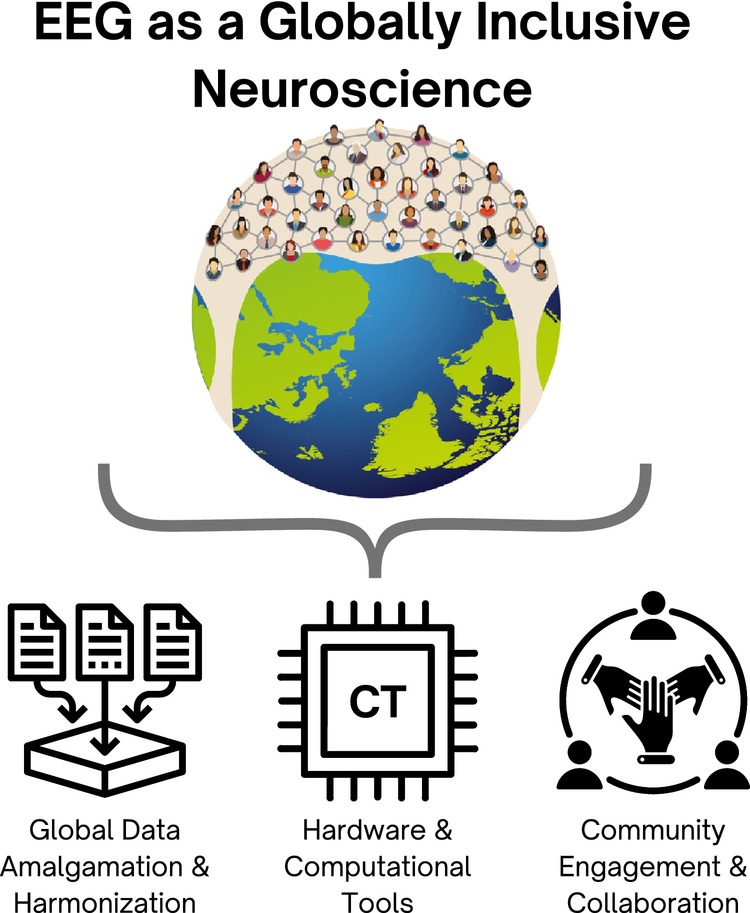

**Abbreviations**
BIDS: Brain Imaging Data Structure
EEG: electroencephalography
ERP: event‐related potential
fMRI: functional magnetic resonance imaging
GBHI: Global Brain Health Institute
GBC: Global Brain Consortium
HarMNqEEG: Harmonized Multinational qEEG Norms algorithm
PPI: patient and public involvement
ReDLat: Multi‐Partner Consortium to Expand Dementia Research in Latin America
WEIRD: Western, Educated, Industrialized, Rich, and Democratic

Much has been written in the social psychological sciences on the problems that emerge from only sampling from Western, Educated, Industrialized, Rich, and Democratic (WEIRD) populations. The fact that most of our understanding of brain structure and function comes from a small segment of the global population is a major cause for concern in neuroscience and brain health research too (Greene et al. 2022). There are clear downstream impacts on translating basic human neuroimaging research into effective interventions and treatments. We propose that scalp‐recorded electroencephalography (EEG), having recently celebrated its centenary (Mushtaq et al. 2024), is one of the most viable tools for delivering a genuinely inclusive and global neuroscience.

EEG signals offer a rich array of analytical possibilities, including, but not limited to, event‐related potentials (ERPs)—time‐locked responses to specific events and time‐frequency decompositions—capturing oscillatory dynamics, connectivity measures, and underlying biophysical mechanisms. Advances in hardware have expanded data collection capabilities, enabling research in diverse environments, including open‐air rural settings, and improving access to underrepresented communities (Vianney et al. 2024). Such efforts have also revealed that “brain clocks” from diverse populations, modulated by physical exposome, gender and disease disparities, socioeconomic inequality, and dementia, can be similar—or even more robustly assessed—with EEG than with fMRI (Moguilner et al. 2024). There has also been a marked rise in open‐source software to support advanced analysis, increasing accessibility for neuroscientists around the globe (Lu et al. 2024).

Yet, despite its potential, the neuroscience community appears skeptical: Many believe we are decades away from EEG being a truly global tool (Mushtaq et al. 2024).

Here, we highlight challenges and suggestions for overcoming key barriers. We propose a multifaceted approach focusing on (i) amalgamation of large‐scale datasets; (ii) technical advances; and (iii) community engagement (Figure 1).

**FIGURE 1:**
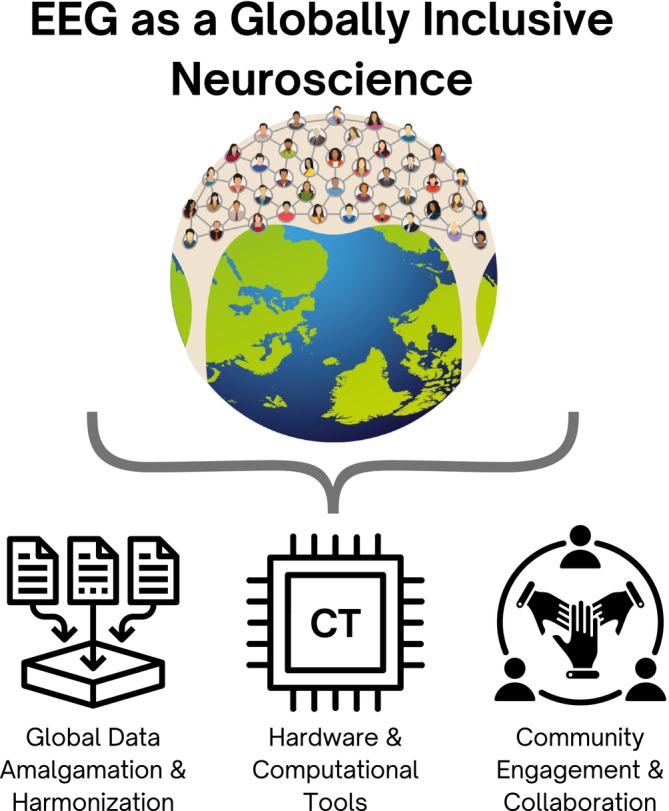
EEG as a globally inclusive neuroscience approach. Key components supporting EEG as a globally inclusive neuroscience tool. The framework integrates hardware and computational tools, community engagement and collaboration, and global data amalgamation and harmonization to advance EEG research worldwide. These elements collectively enable the development of inclusive, scalable, and high‐impact neuroscience applications across diverse populations.

Many EEG datasets are already available from different parts of the world. But they are nothing if not fragmented. Standardization, for example, through Brain Imaging Data Structure (BIDS), although increasingly common, is not universal. And when the same tasks are employed to examine the same purported constructs, substantial variation in task parameters can arise, which may be further compounded by language differences—from instructions to stimuli presentation. Benchmark datasets offer a foundation for cross‐site comparisons (Kappenman et al. 2021). However, expanding their use beyond individual laboratories and across diverse settings will help progress the field.

On signal processing, advances in data harmonization can increase the interoperability and utility of shared data. The Harmonized Multinational qEEG Norms (HarMNqEEG) algorithm (Li et al. 2022) developed by members of the Global Brain Consortium (GBC) is an example of such an effort. They introduced a Riemannian approach to harmonizing cross‐spectral tensor data, connecting 1564 datasets from 14 sites collected over 50 years. Computational advancements are also addressing data diversity challenges. Data augmentation and deep learning techniques can help reveal biophysical mechanisms, model perturbations, biological noise, and multimodal priors (Ibanez et al. 2024), whereas synthetic datasets can improve group representation, boosting sensitivity, and generalizability (Moguilner et al. 2024). Generative biophysical EEG models can address heterogeneity and incorporate multiple priors, making them more robust to individual differences (Coronel‐Oliveros et al. 2024). More broadly, probabilistic frameworks including Bayesian and Markov models enable continuous updating of predictions as new individual data becomes available (Ibanez et al. 2024). Together, these advances pave the way for large‐scale harmonized databases, strengthening statistical and computational power to better understand population differences.

Hardware limitations remain a significant barrier to global EEG research. Although portable EEG systems enhance accessibility, affordability—particularly in low‐ and middle‐income countries—remains a major challenge. Open‐source and commercial systems offer promise, but balancing cost, electrode density, and signal quality is difficult (Niso et al. 2023). These trade‐offs are especially pronounced in large‐scale studies and fieldwork, where fewer electrodes are used to meet logistical and resource constraints. Beyond cost, operational complexity and participant‐specific challenges further limit feasibility. Many EEG systems require specialized training, restricting their use where trained technicians are unavailable. Additionally, obtaining high‐quality recordings remains difficult for individuals with specific hair types, as standard EEG caps often struggle to maintain electrode contact, leading to lower signal quality and frequent participant exclusion. Innovative solutions, such as comb‐shaped “fingered” electrodes, show promise in improving signal quality by better adapting to textured hair (see Choy et al. 2022 for a review of possible solutions). To enable broader adoption, EEG platforms must simplify setup, automate calibration, and provide intuitive interfaces that allow data collection with minimal expertise.

Direct engagement with underrepresented communities is essential for inclusive neuroscience. Most EEG research relies on convenience sampling from affluent, highly educated populations in the Global North. We now have the opportunity to take EEG out to historically underrepresented communities. This will require culturally sensitive research practices (e.g., how will you collect data from a Muslim woman wearing a hijab?) and community engagement strategies (e.g., how will you encourage participants to take part in studies when they have more pressing priorities affecting their daily lives?) and must be complemented by robust ethical safeguards, including transparent data governance, informed consent tailored to local norms, and mechanisms for community oversight. Frameworks such as the TRUST Code emphasize equitable research partnerships while integrating Patient and Public Involvement (PPI) principles can help foster trust and support long‐term collaboration.

Several initiatives are already tackling these challenges. Large‐scale EEG acquisition efforts in India and Tanzania (Vianney et al. 2024) and international collaborations like GBC, ReDLat, the Global Brain Health Institute (GBHI), EuroLad‐EEG, and #EEGManyLabs (Pavlov et al. 2021) demonstrate how multisite research can enhance inclusivity. The GBC has been building networks to support neuroscientists in the Global South. ReDLat, EuroLad‐EEG, and GBHI are illustrating the impact of macrosocial factors on brain health (Moguilner et al. 2024). The #EEGManyLabs initiative is showcasing the power of multisite collaboration, providing a model for achieving the scale necessary to address complex questions about brain function. To build on these foundations, sustained investment is needed in training, infrastructure, and multisite collaboration must become the norm, not the exception.

## Author Contributions

**Faisal Mushtaq:** conceptualization, writing – original draft, writing – review and editing. **Agustín Ibáñez:** conceptualization, writing – original draft, writing – review and editing.

## Conflicts of Interest

The authors declare no conflicts of interest.

### Peer Review

The peer review history for this article is available at https://www.webofscience.com/api/gateway/wos/peer‐review/10.1111/ejn.70078.

## Data Availability

There are no pertinent data associated with this commentary article.
